# A Vision Based Top-View Transformation Model for a Vehicle Parking Assistant

**DOI:** 10.3390/s120404431

**Published:** 2012-03-30

**Authors:** Chien-Chuan Lin, Ming-Shi Wang

**Affiliations:** Department of Engineering Science, National Cheng Kung University Taiwan, No.1, University Road, Tainan City 701, Taiwan; E-Mail: mswang@mail.ncku.edu.tw

**Keywords:** top-view transformation, bird's eye view, inverse perspective mapping

## Abstract

This paper proposes the Top-View Transformation Model for image coordinate transformation, which involves transforming a perspective projection image into its corresponding bird's eye vision. A fitting parameters searching algorithm estimates the parameters that are used to transform the coordinates from the source image. Using this approach, it is not necessary to provide any interior and exterior orientation parameters of the camera. The designed car parking assistant system can be installed at the rear end of the car, providing the driver with a clearer image of the area behind the car. The processing time can be reduced by storing and using the transformation matrix estimated from the first image frame for a sequence of video images. The transformation matrix can be stored as the Matrix Mapping Table, and loaded into the embedded platform to perform the transformation. Experimental results show that the proposed approaches can provide a clearer and more accurate bird's eye view to the vehicle driver.

## 1. Introduction

The parameters of interior and exterior cameras are necessary to process images for coordinate transformation and calibration, but it is not easy obtain to those parameters, video frames especially.

Parking a vehicle safely is an important issue, but not an easy task for some drivers. Installing sensors at the rear of the vehicle is helpful when driving the vehicle in reverse. A video-based auxiliary system provides the driver with images from the rear of the vehicle, the driver can know the environment of the desired parking area very well, and further actions then depend on driver decisions. In recent years, manufacturers have equipped many vehicles with rearward-facing cameras to improve driving safety. Most of these cameras only display captured images on an in-vehicle screen. The driver cannot easily judge the depth and positioning from these images. For example, the images do not convey the distance between the vehicle and any obstacles located behind the vehicle.

Therefore, this study proposes a TVTM (Top-View Transform Model) approach to apply to a video-based auxiliary parking assistant system that provides drivers with a clearer bird's eye view of the rear-end area around a vehicle. The main contribution of this paper is to propose a coordinate transformation model that does not need any interior and exterior camera parameters and can adapt the setup position of the camera. In addition, the proposed approach could speed up the processing performance on an embedded platform.

The remainder of this paper is organized as follows: Section 2 surveys some related works about perspective transformation. Section 3 describes first the proposed top-view transform model approaches to a single image and then how to extend them to process video frames is explained. The experimental results and some discussions are given in Section 4. Finally, a brief conclusion is provided in Section 5.

## 2. Related Works

Currently, most video-based vehicle assistant systems focus on the forward direction of the vehicle. Schreiber [1] proposed a robust scheme to detect and track lane markings to delimit road boundaries, using a forward-looking single camera. McCall and Trivedi [2] designed a video-based driver's assistant system to estimate and track lane markings on the road. Jung [3] used a monocular vision based parking-slot-markings recognition algorithm to find the parking space, which transforms source images into a bird's eye view image and uses the Hough transform to find the edge of parking slot-lines. Jung [4] used a light stripe projection based free parking space recognition method to overcome the common drawbacks of existing vision based target position designation methods in dark indoor parking sites. Scheunert [5] proposed a park-slot finding auxiliary system, which combines a camera and a non-visual sensor to find any available park-slot in the area while the vehicle is passing by. Liu [6] proposed a bird's eye view vision system for vehicle surrounding monitoring which installed six cameras on a vehicle and stitched the views from those six cameras. Ehlgen [7] combined the images of four cameras to obtain a view of the whole surrounding area of vehicle with the purpose of eliminating the driver's blind spots.

The inverse perspective mapping scheme is another method for obtaining a bird's eye view of the scene from a perspective image. The inverse perspective mapping technique can also be used to removes the perspective distortion caused by the perspective projection of a 3D scene into a 2D image. In general, each proposed inverse perspective mapping method uses different transformation mechanisms. Based on the linear mapping of homogeneous coordinates, Muad [8] addressed the mathematical theory of inverse perspective mapping. In another study, Tan, Dale, Anderson and Johnston [9], provide the basic geometrical transformation structure and formulas for inverse perspective mapping. The reconstructed “median plane” links the two transformed objects. This method depends on three parameters: θ_0_, the rotation angle between the image plane and the median plane; Z_0_, the distance between the image plane and the median plane; and c_0_, the distance between the image plane and the X axis on the world coordinate system. This method can be applied when these parameters are available. Unfortunately, these three parameters are unknown in most cases. Bertozzi [10] proposed a heuristic search method to find homologous points. Some researchers have also used the vanishing point phenomenon to calculate the inverse perspective mapping model [11,12].

## 3. Top-View Transformation Model

**Figure 1.**
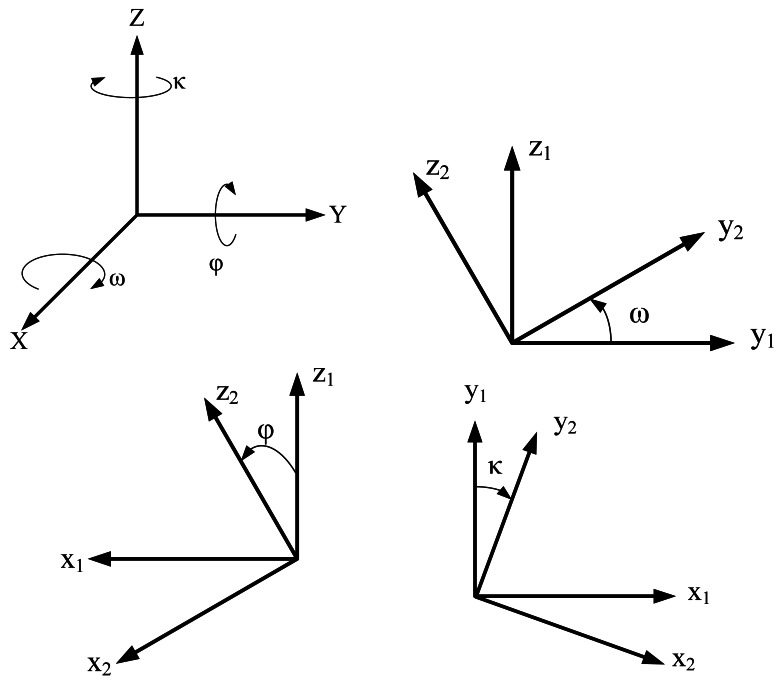
The rotation model on X, Y and Z coordinate axes.

The objective of the proposed top-view transformation model is to provide a clearer top-view image than traditional inverse perspective mapping. Figure 1 shows the rotation model for each of the coordinate axes in a 3D Cartesian coordinate system. Assume that the rotation occurs in a clockwise direction (the X-axis is pointing out) and the X-axis, Y-axis, and Z-axis rotate ω, φ, and κ degrees, respectively. Then the rotation matrices, in homogeneous coordinates form, for X-axis, and Y-axis, Z-axis can be represented as Rω, Rφ, and Rκ respectively, as the Equation (1). In Rω matrix, the coordinate X-axis is invariant and Y-axis and Z-axis rotated ω angle. Similarly, in Rφ matrix, the coordinate Y-axis is invariant, and in Rκ matrix, the coordinate Z-axis is invariant:

(1) $$R_{\omega }=\left[\begin{array}{cccc} 1 & 0 & 0 & 0\\ 0 & \cos\omega & \sin\omega & 0\\ 0 & -\sin\omega & \cos\omega & 0\\ 0 & 0 & 0 & 1 \end{array}\right];R_{\varphi }=\left[\begin{array}{cccc} \cos\varphi & 0 & -\sin\varphi & 0\\ 0 & 1 & 0 & 0\\ \sin\varphi & 0 & \cos\varphi & 0\\ 0 & 0 & 0 & 1 \end{array}\right];R_{\kappa }=\left[\begin{array}{cccc} \cos\kappa & \sin\kappa & 0 & 0\\ -\sin\kappa & \cos\kappa & 0 & 0\\ 0 & 0 & 1 & 0\\ 0 & 0 & 0 & 1 \end{array}\right]$$

Homographic mapping method [13] is used to illustrate the relationship between two different views of the same real world scene. Let p and p′ be the corresponding projected image points on the image plane of two different views of the same point located in the 3D real world coordinates system. Assume the coordinates of this pair of matching points, p and p′, in inhomogeneous form are denoted as (*x*_1_, *y*_1_)*^T^* and (*x*_2_, *y*_2_)*^T^*, respectively, where T denotes the vector transpose. Without loss of generality, the homogeneous coordinate representations of these two points are (*x*_1_, *y*_1_, *z*_1_)*^T^* and (*x*_2_, *y*_2_, *z*_2_)*^T^*, respectively. The homographic mapping of the two points is then a planar projective transformation, and can be expressed as Equation (2) for a homogeneous form. This homographic mapping is a linear transformation. The nonsingular 3 × 3 matrix *H* is called the homogeneous transform matrix. To obtain the inhomogeneous form representation (*x*_2_, *y*_2_) of the vector (*x*_1_, *y*_1_, *z*_1_)*^T^* of Equation (2) and Equation (3) are adopted. A homographic transformation can also be used to remove the projective distortion due to the perspective projection from a 3D scene into a 2D plane image. The main problem is how to select the points, the (*x*_1_, *y*_1_, *z*_1_)*^T^* vectors applied in Equation (2), to perform the transformation, as different selected points produce different solutions:

(2) $$\left(\begin{array}{c} x_{2}\\ y_{2}\\ z_{2} \end{array}\right)=\left[\begin{array}{ccc} h_{11} & h_{12} & h_{13}\\ h_{21} & h_{22} & h_{23}\\ h_{31} & h_{32} & h_{33} \end{array}\right]\left(\begin{array}{c} x_{1}\\ y_{1}\\ z_{1} \end{array}\right)\Rightarrow {\left(\begin{array}{c} x_{2}\\ y_{2}\\ z_{2} \end{array}\right)}^{\prime }=H\left(\begin{array}{c} x_{1}\\ y_{1}\\ z_{1} \end{array}\right)$$

(3) $$\begin{array}{l} x_{2}=\frac{x_{1}}{z_{1}}=\frac{h_{11}x_{1}+h_{12}y_{1}+h_{13}}{h_{31}x_{1}+h_{32}y_{1}+h_{33}}\\ y_{2}=\frac{y_{1}}{z_{1}}=\frac{h_{21}x_{1}+h_{22}y_{1}+h_{23}}{h_{31}x_{1}+h_{32}y_{1}+h_{33}} \end{array}$$

### 3.1. The Camera Model

The camera model structure is the basic theory and concept for developing a computer vision application. In this application, the 3-D real world coordinate system is adopted by assuming that a Right Hand Cartesian coordinate system is used, the Y-axis extends out in the forward direction of the vehicle; the X-axis points out to the right of the vehicle; and the Z-axis is perpendicular to the ground. The camera used to capture the rear-end images of the vehicle is mounted at the center of the bumper of the vehicle.

**Figure 2.**
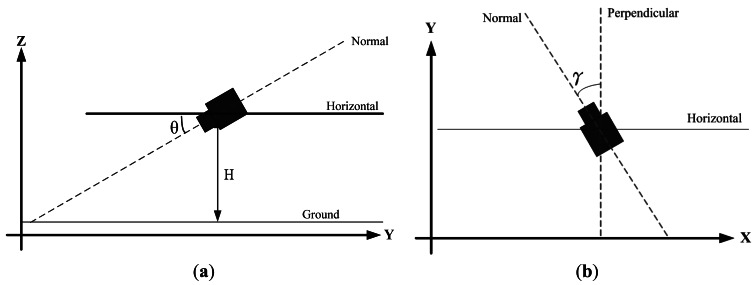
The camera model used in this paper. (**a**) View of the Y-Z plane for height and tilt; (**b**) View of the X-Y plane.

Figure 2 shows two views of the camera model used in the proposed method. Figure 2(a) shows the model viewed from the Y-Z plane (projected on the Y-Z plane), where H denotes the distance between the camera and the ground and the tilt angle θ is defined as the angle between the Normal line (the optical axis) of the camera and the Horizontal line. Figure 2(b) shows the model viewed from top to the ground, where the angle γ represents the pan angle of the camera in the coordinate system. The angle γ can be viewed as the angle between the projection line of the camera's optical line on the X-Y plane and the Y-axis. If the pan angle γ = 0, the projection line of the camera's optical line on the X-Y plane is parallel with the Y-axis. In this case, the image captured by the camera is just the perspective projection image of the real world at the rear of the vehicle. If γ is not zero, the captured image is skewed to the left or right of the rear of the vehicle.

**Figure 3.**
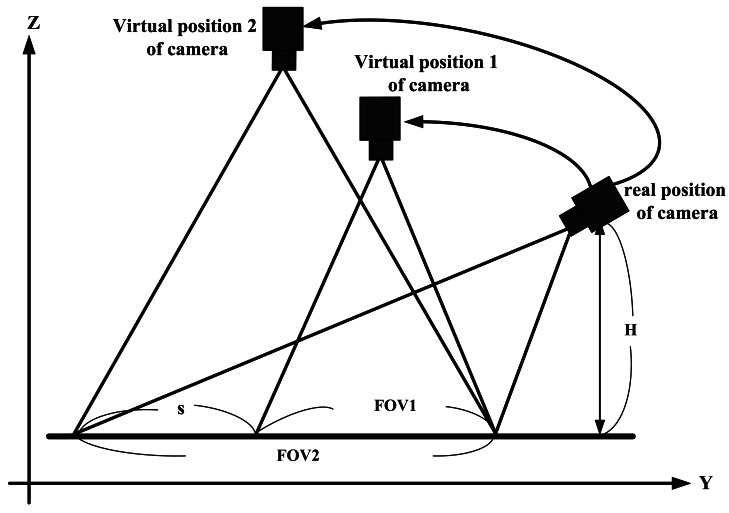
Related positions of the camera in TVTM that view from the Y-Z plane.

A camera's FOV (Field Of View) defines the pyramid field. The apex located at the center of the image plane of the camera, of the real world in front of the camera that can be captured by the camera and projected onto its image plane. The amount of the scene within this pyramid space region that will be projected onto the camera image plane depends on both the FOV and the “block plane” in front of the camera. In this application, the “block plane” is the ground. The truncated pyramid is called a frustum. Due to the camera can be located at different height and/or with different tilt angle θ, the frustum is skewed depending on which conditions applied. For simplifying the description, in Figure 3, the effective “block plane” projected on the Y-axis is used to denote the effective FOV for different case, for example, FOV1 is used to represent the frustum for the camera located at virtual position of camera 1.

Figure 3 illustrates the same camera located at three different positions, showing the effective FOV in the direction of the Y-axis. The real position one is denoted the camera that is installed on the car with distance H above the ground and with effective FOV, FOV2. If the camera is located at virtual position 1, the height of the camera is higher than H but less than that of virtual position 2, which reduces the effective FOV of the camera at virtual position 1 to FOV1. If the camera is located at the virtual position 2, then its effective FOV is as same as that of the real camera position. In this study, the basic requirement of the equipment is to provide the driver with clear images that represent the reality behind the car to assist with car parking. Therefore, it is desired to show the image captured closer to the rear of the vehicle and include full information about the parking spot. From this viewpoint, it is fitter to show the driver the image obtained by effective FOV1 instead of FOV2 that just displays the area that the driver is most concerned. FOV1 can also display a large and clear image on the screen.

In the following subsection, the proposed top view transform model will be described. The TVTM transforms the image captured at the real camera position into an image equivalent to that captured at the virtual camera position 2, and both of these images are displayed on the same size (due to the two cases with the same effective FOV). Figure 3 shows that, for practical applications, the three images with different effective FOV's share the same end point, which corresponds to the nearest end of the car where the camera captures its image. As illustrated in Figure 3, the effective FOV1 can be reduced from that of FOV2 by discarding the portion denoted by ***s***. This means that FOV1 = FOV2 − ***s***.

### 3.2. Top-View Transformation Model

Based on the rotation transformation described above Equation (1) in Section 3, and the camera model in the Section 3.1, the TVTM formula can be described as Equation (4), where (x, y) are the original image coordinates, and (x*, y*) are the destination image coordinates, *H* is the distance between the camera and ground, *f* is the focal length of the camera, and *θ* is the camera tilt angle. Equation (4) shows that the transformed component values of x* and y* may be less than or equal to zero. To ensure that the transformed component values of x* and y* are not the less than zero and keep the condition that the coordinate point on the original source image is mapped into the coordinate point in the destination image coordinate. In Equation (5) a constant *d* is defined as |*H*(sin*θ* + cos*θ*)/(*f*sin*θ* − cos*θ*)|. This means that the coordinate point in the original source image has been mapped into the point of the destination image coordinate system. Equation (5) gives the proposed formulas for the TVTM transformation:

(4) $$x^{*}=H\frac{x\sin\theta +f\cos\theta }{-y\cos\theta +f\sin\theta };y^{*}=H\frac{y\sin\theta +f\cos\theta }{-y\cos\theta +f\sin\theta }$$

(5) $$x^{*}=H\frac{x\sin\theta +f\cos\theta }{-y\cos\theta +f\sin\theta }+d,y^{*}=H\frac{y\sin\theta +f\cos\theta }{-y\cos\theta +f\sin\theta }+d,\text{where}\;d=\left|\frac{H(\sin\theta +f\cos\theta )}{f\sin\theta -\cos\theta }\right|+1$$

As previously stated, to provide the driver with clearer images that represent the reality behind the car, it is better to only show images captured close to the rear end of the car, including the full information about the parking spot. This goal can be reached by first discarding the portion of the captured image that represents the far side of the camera's field of view. The upper side of the image represents the far end of the camera's field of view. The upper portion above the dashed horizontal line can be discarded without affecting the information provided to the driver. This also improves the processing time and helps provide a clearer image. The area of the image to be discarded depends on the value of parameter ***s***. Here, the parameter ***s*** is a dynamic parameter that is used to tune the effective FOV1 field.

**Figure 4.**
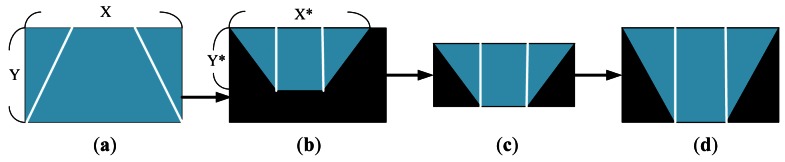
Procedures of TVTM. (**a**) Source perspective image; (**b**) Image after TVTM transformation; (**c**) Image after discard non-information part of (b); (**d**) Image resized from (c).

Figure 4 depicts the TVTM processing flow by showing the processed result of each step for easy understanding. Figure 4(a) shows a source perspective image containing the two side line sections of a parking spot. Figure 4(b) shows the corresponding image after TVTM transformation of the image in Figure 4(a). In Figure 4(b), only the rectangle region bounded by X* and Y* represents the information contained in the original image. This rectangle region with side lengths X* and Y* is then extracted in Figure 4(c). The image in those figures is then resized to the same size as the original image.

### 3.3. Optimal Searching Algorithm for TVTM Parameters

Based on the provided source image, this subsection estimates the parameters of *H, f* and *θ* used in Equation (5). For an installed image captured system that can provide the installed system values of *H, f* and *θ* as the input initial values for the proposed searching algorithm to find the fittest parameters set used for the given source image. Even when the installed system values of *H, f* and *θ* are not provided, the proposed searching algorithm can determine the fittest parameter values of *H, f* and *θ* for the given source image. However, this requires significantly more processing time.

**Figure 5.**
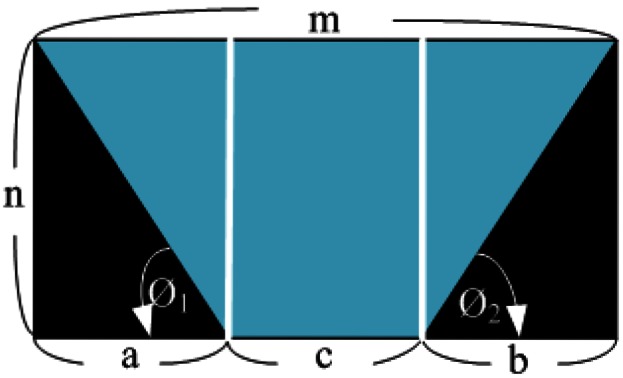
Example of TVTM transformed image.

Before finding the three parameters, *H, f* and *θ* used in Equation (5) from a given source image, let us consider some transformed results under different parameter values. Figure 5 gives one possible TVTM transform result. Assume that the image size is *m* × *n* pixels. The horizontal axis and the vertical axis in Figure 5 point out in the rightward direction and the backward direction, respectively. The colored trapezoid area in Figure 5 represents the same content included in the source image. Let ***a, b***, and ***c*** be the length of the three segments as shown in Figure 5. Then, if ***a*** = ***b*** is true, then the angle φ_1_ is equal to the angle φ_2_ Equation (6) illustrates the values of φ_1_ and φ_2_:

(6) $$\phi_{1}=\cos^{-1}(\frac{a}{\sqrt{n^{2}+a^{2}}}),\phi_{2}=\cos^{-1}(\frac{b}{\sqrt{n^{2}+b^{2}}})$$

**Figure 6.**
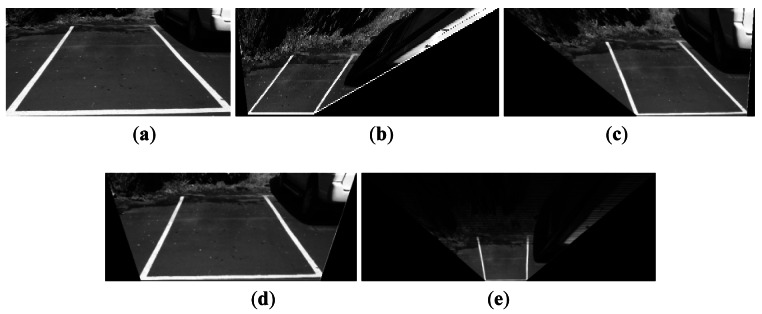
Example of TVTM transformed images with different parameter values of H, f and θ; (**a**) Source image; (**b**) Skewed to left side; (**c**) Skewed to right side; (**d**) Too wide in the bottom side; (**e**) Too short in the bottom side.

Figure 6(b–e) shows four TVTM transformation results from the same source image shown in Figure 6(a) with varying parameter values. The transformed result shown in Figure 6(b) is skewed to the left. In this case, the condition ***a*** < ***b*** is satisfied and it is caused by both of the parameters *θ* and *H* with too small value. Figure 6(c) shows the case that the transformed result is skewed to the right. It results that ***a*** > ***b*** is satisfied and the reason is that both of the parameters *θ* and H are with too large value. Figure 6(d) gives the transformed result that the conditions ***a*** = ***b*** and ***c*** > ***b*** are met. This case is caused by a large value of the parameter *f*. Figure 6(e) illustrates the transformed result under the condition that ***a*** = ***b*** and ***c*** < ***b*** are satisfied. It results from too small a value of the parameter *f*.

In order to provide the driver with the best quality of the transformed image, according to our experience, the condition of Equation (7) must be satisfied. Under this condition, the transformed image is always displayed at the center area of the screen and the entire image contained in the original source image, especially for these providing the parking slot area is kept on the transformed version. Due to the fact the data is processed in digital form; the condition of Equation (7) is not always satisfied. To conquer this problem, the testing condition was modified to test if all of the three conditions as Equation (8), where ε, and ε′ are given very small positive values; ω and ω′ are given interval values, are satisfied:

(7) a=b=c=(m/3)

(8) $$\left|a-b\right|<ɛ,\omega >c/m<\omega^{\prime },\text{and}\;\omega^{\prime }=\omega +ɛ\prime$$

**Figure 7.**
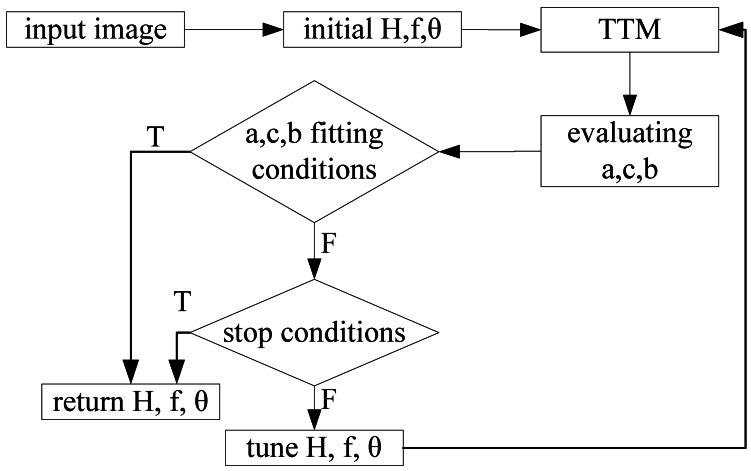
The flow chart of the fittest algorithm for parameters of TVTM.

Figure 7 illustrates the flow chart for obtaining the fittest values of *H, f* and *θ* of the input source image. This flow chart works as follows: first, given a set of initial values for *H, f* and *θ*, then we perform the TVTM transformation via Equation (5). Next, the values of ***a, b***, and ***c*** are calculated based on the transformed image, the output of TVTM transform. Then the relationships among the values of ***a, b***, and ***c*** are examined. If the predefined condition is satisfied, it means that the ideal condition ***a*** = ***b*** = ***c*** = **(m / 3)** is met, then output the fittest values of *H, f* and *θ*.

If the predefined condition is not satisfied then we must refine the *H, f* and *θ* values accordingly. Then, we use the refined parameters' values to perform the TVTM transformation and enter the next loop searching for the fittest parameters' values of the input source image. To avoid trapping the algorithm in infinite loops, a stop condition can be set, for example, the algorithm ends if it loops more than N times, where N is a given number. The evaluation process of **a, c, b** is to evaluate and obtain the new values of **a, c**, and **b**. Then the conditional testing block is used for finding the relationships among ***a, b***, and ***c*** and the parameters again until they match the fitting conditions or the stop conditions. This algorithm keeps the results of H, f and ***θ*** along with some setup position of camera, for example, height, tilted angle and rotation angle. Therefore, the TVTM approach would be an auto adaptive calibration model.

### 3.4. Implement TVTM into the Embedded Platform

**Figure 8.**
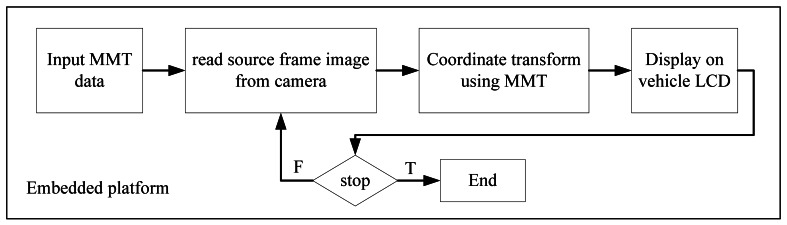
Processing flow of TVTM on an embedded platform with LCD screen.

It is relatively easy to extend the single image processing scheme above to video frame images. Assume all of the images in a video image have the same resolution and the parameter values of *H, f*, and *θ*, remain unchanged for all video frames. Under this condition, only the parameter values of the first image frame must be estimated. The following frames can use the same parameter values evaluated from the first frame. This means that only the first image frame of the video image needs to execute the parameter searching algorithm described in the previous subsection. All information obtained from the coordinate transformation of a source frame image into the TVTM transformed image is stored in a matrix, called the Mapping Matrix Table (MMT), for later use. Using this approach reduces the processing time for subsequent image frames. Since the transformation parameters are saved in the MMT, it can be implemented on a low computing power platform, such as the embedded platform. Figure 8 illustrates the processing flows of TVTM on the embedded platform. The first image frame of a video image is processed by a personal computer to obtain its TVTM transformation data, and this data is stored in the MMT matrix. The MMT matrix is then loaded and saved into the storage of the embedded platform. The embedded platform uses the MMT data to execute the TVTM transformation for the input image frames. The experiments in this study has been implemented an embedded platform.

### 4. Experimental Results and Discussion

**Figure 9.**
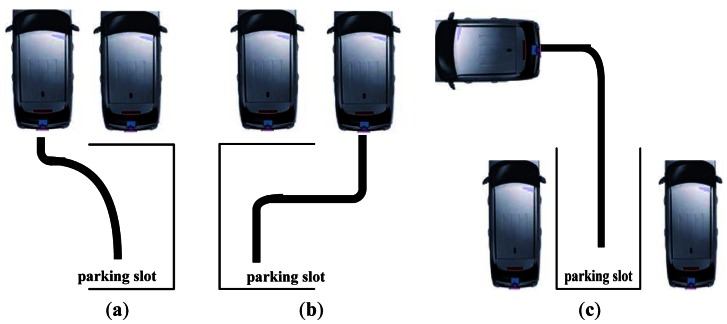
The considered parking cases; **(a)** Right rear; **(b)** Left rear; **(c)** Backward.

The experiment environment was set up as follows: the parking spot locations included the right rear, left rear and backward as Figure 9 indicates. Three different camera tilt angles, θ = 40°, θ = 45°, and θ = 50°, were considered for each scenario. The camera was installed on the back bumper of the vehicle at a height of 80 centimeters above the ground. The camera panned angle γ is small and assumed to be zero. The source video image is color, with 720 × 480 resolutions. The parameter ***s*** used in TVTM to discard a portion of the image was fixed to 100 pixels. The experiments were first conducted on the MATLAB software package. Finally, the proposed scheme has been implemented in c-language and run under an embedded platform.

### 4.1. Evaluation of TVTM

The camera used to capture video images was installed above the rear bumper of the vehicle and equipped with a 124° wide field lens at a height of 80 cm above the ground. Therefore, each video image shows the bumper of the vehicle in the bottom area. The frame rate is 30 frames per second. As indicated in Section 3, the first image frame of the captured video image is extracted and processed to derive the transformation matrix. The transformation matrix will be stored in the MMT matrix that used to process the following image frames.

**Figure 10.**
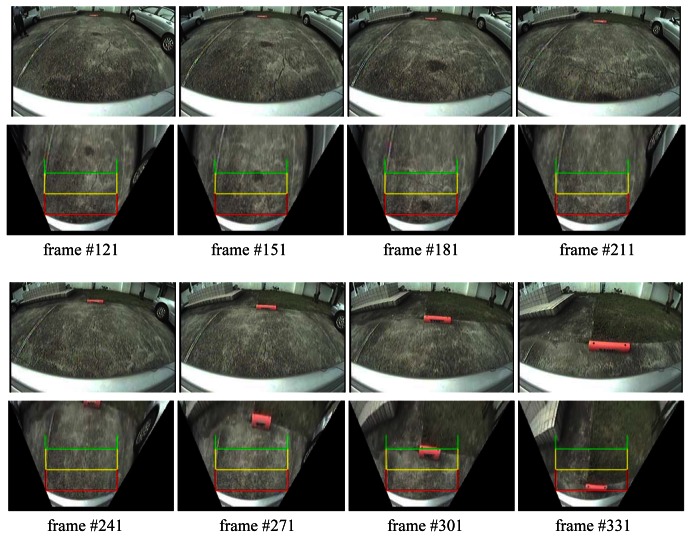
Experimental results for direct backward parking with θ = 45°. In each image frame, the top one is the source image and its TVTM result is shown at the bottom.

Figure 10 shows a sequence of eight sample video frames. In this scenario, the parking space is located just behind the vehicle. Those sub figures show two images for each sample image frame; the upper one is the original source image, and the lower one is its corresponding image transformed by the TVTM transformation. The broad curved white line located at the bottom of the image denotes the rear bumper of the vehicle. The transformed image also contains three auxiliary lines, marked with the “ㄩ ” symbol, and colored red, yellow, and green, to help the driver determine the relative position of obstacles at the rear of the vehicle. If the image shows that an obstacle will touch the red line, it means that an obstacle will touch the rear bumper of the vehicle. The yellow line indicates a distance of about 60 cm from the rear end of the vehicle, while the green line represents a distance of about 120 cm. The rear bumper and auxiliary lines are always on the same position for each TVTM result frame image. They can help the driver recognize and understand the relative distance between the vehicle and obstacles.

To change the tilt angle of the installed camera will change the depth of the scene to be included in the captured image. In this experiment, three different tilt angles are considered θ = 40°, θ = 45°, and θ = 50°. As shown in Table 1, the depths of the scene to be included in the captured image away from the rear bumper of the vehicle for θ = 40°, θ = 45°, and θ = 50° are more than 10 meters, more than 9 meters, and about 5 meters, respectively.

Table 1 also gives the corresponding effective depths for these three cases of the transformed TVTM image. Based on the data shown in Table 1, the final displayed image can provide the driver the scene of 2.5 meters depth away from the rear bumper of the vehicle. The tilt angle θ is set to 45° in our final system.

### 4.2. Frame Rate Estimation on the Embedded Platform

This study evaluates different approaches of the TVTM transformation on the embedded platform. Executing the TVTM transformation directly on the embedded platform requires 113 μs/frame, which allows 9 frames/s to be processed. The approaches fail to meet the practical requirement of 15–30 frame/s. Pre-calculating and storing the transformation parameters and matrix for later use dramatically reduces the processing time. Performing the TVTM transformation on the embedded platform via MMT matrix, the performance is 36 ms/frame. The processing rate for this approach is 27 frames/s.

### 4.3. The Result Evaluation

The similarity evaluation was performed using Normalized Cross Correlation (NCC) [14]. The NCC is a cosine-like correlation coefficient. Equation (9) lists, r(*u*,*v*), the function of correlation coefficient. If the value of NCC is closer to 1, then it presents the more similar between two images:

(9) $$r(u,v)=\frac{\underset{xy}{\text{∑}}\left[f(x,y)-{\bar{f}}_{u,v}\right]\left[t(x-u,y-v)-\bar{t}\right]}{{\left\{{\sum_{xy}\left[f(x,y)-{\bar{f}}_{u,v}\right]}^{2}{\sum_{xy}\left[t(x-u,y-v)-\bar{t}\right]}^{2}\right\}}^{0.5}}$$

where *f* is source image, *t̄* is the mean of the TVTM transformed image and *f̄_u, v_* is the mean of *f*(*x, y*) in the region under the TVTM transformed image.

**Figure 11.**
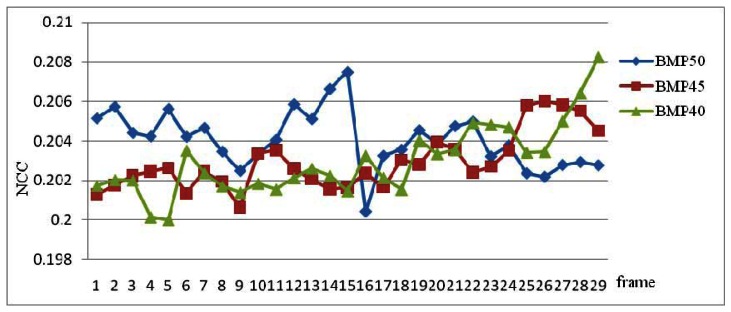
The chart of NCC values of 29 sub-frames of backward middle parking video with θ = 50, 45 and 40 degree.

Figure 11 shows the NCC values from 29 sub-frames source image and TVTM transformed image of backward middle parking video with θ = 50, 45 and 40 degree. The average of NCC of BMP50 (Backward Middle Parking with θ = 50) is 0.2041, BMP45 (Backward Middle Parking with θ = 45) is 0.2043, and BMP40 (Backward Middle Parking with θ = 40) is 0.2030. The total average of NCC is 0.2038.

**Figure 12.**
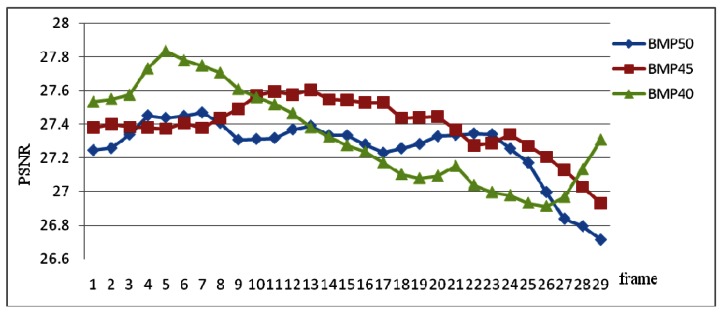
The chart of PSNR values of 29 sub-frames of backward middle parking video with θ = 50, 45 and 40 degree.

The Peak Signal-to-Noise Ratio (PSNR) is used to evaluate noise between two images. Equation (10) illustrates the function of Mean Squared Error (MSE) that is one of many ways to quantify the difference between two images, where I (I, j) and K (I, j) are two image matrices. Based on MSE, the PSNR presented as Equation (11), where MAX is the maximum of image and its value equals 255. Figure 12 shows that the PSNR values from 29 sub-frames source image and TVTM transformed image of backward middle parking video with θ = 50, 45 and 40 degree. The average of PSNR of BMP50 is 27.2615, BMP45 is 27.3322, and BMP40 is 27.3346. The total average of PSNR is 27.3094:

(10) $$\text{MSE}=\frac{1}{mn}\sum_{i=0}^{m-1}\sum_{j=0}^{n-1}{\left[I(i,j)-K(i,j)\right]}^{2}$$

(11) $$\text{PSNR}=10\times \log_{10}(\frac{MAX_{I}^{2}}{\text{MSE}})$$

### 4.4. Comparison with other IPM Methods

Usually, the image quality is not good after inverse perspective mapping [8,9,11,12] transformation. The proposed approach is clearer than those. Table 2 lists the NCC and PSNR value comparison between the proposed method and the other inverse perspective mapping schemes. The average NCC of the proposed method is the largest one of those listed in Table 2. The average PSNR of the proposed method is less than [8] and [9], but our NCC is twice that.

### 4.5. Discussions

The proposed TVTM method clipped the vague and remote image part. According to the data in Table 2 and the transformed images, the view of the TVTM result image is clearer than traditional IPMs [8,9,11,12] and the processing time is less. The distance of view is shorter than traditional IPMs. The clipped image part, need not be displayed, because it is not clear. The proposed TVTM method is stable. According to the results of NCC and PSNR, there is little difference between the maximum and minimum of NCC and PSNR, respectively.

The exterior orientation parameters of camera are not measured easily, and the interior orientation parameters of camera must usually be calibrated. The proposed fitting parameter searching algorithm is used to obtain the optimal fitting parameters of TVTM, which can tune those parameters automatically to adapt the changes of the camera setup angle and height. On the other hand, the proposed algorithm does not need to calibrate the exterior or interior orientation parameters of the camera. The proposed algorithm can obtain the parameters of TVTM to do the coordinate transformation automatically.

The Mapping Matrix Table method is used to speed up the system performance on an embedded platform, but the MMT scheme is only allowed for the case where the parameters of TVTM are fixed. If the parameters of TVTM have been changed, the MMT data must be produced again. The capabilities of the proposed approach are as follows.

- The proposed approach could easy run on an embedded platform with high computing performance for a vehicle parking assistant system.
- The transferred image is clearer than the other IPM methods.
- The parameters of the proposed transform model could be found and tuned automatically.
- The proposed approach does not need to calibrate the exterior and interior orientation parameters of camera.

The limitations of the proposed approach are as follows:

- In order to provide a good view to the driver, the position of the camera is fixed. For example, the camera is installed on the back bumper of the vehicle.
- The MMT data must be reproduced when the TVTM parameters are changed.

## 5. Conclusions

This paper proposes a Top-View Transform Model approach to transform a perspective projection image into its corresponding bird's eye vision. We have applied this technique to provide the driver with a clearer image of the area behind the car. The proposed searching algorithm for TVTM parameters does not need the exact interior and exterior orientation parameters of camera.

To speed up the processing rate to meet practical requirements, this study proposes the parameter value ***s*** to determine how much of the original image should be included in the final bird's eye view image. The TVTM transformation matrix is stored as the Matrix Mapping Table, and can be loaded into the embedded platform to transform video images based on the embedded platform.

Experimental results show that the proposed approaches can provide clear and accurate bird's eye view vision to the vehicle driver. A prototype system based on the proposed approaches was implemented on an embedded platform. In our future work, the proposed approach will be applied to solve various problems for vehicle applications, such as lane departure, providing the vehicle's surrounding view, and eliminating driver's blind spots.

## Figures and Tables

**Table 1.** The effective depth of source image and its TVTM image.

	θ = 40°	θ = 45°	θ = 50°

source image	>10.0 m	>9.0 m	5.0 m
TVTM transformed image	3.5 m	2.5 m	1.5 m

**Table 2.** The NCC and PSNR of references and our proposed method.

	[8]	[9]	[11]	[12]	Proposed method

NCC	0.1013	0.1046	0.1285	0.1940	0.2038
PSNR	27.5253	27.5319	25.0977	24.9304	27.3094
